# The role of post-translational modifications in parvovirus life cycle

**DOI:** 10.3389/fvets.2025.1634345

**Published:** 2025-07-04

**Authors:** Peng Liu, Liqin Yang

**Affiliations:** School of Ecological Engineering, Guizhou University of Engineering Science, Bijie, China

**Keywords:** parvovirus, phosphorylation, ubiquitination, SUMOylation, glycosylation

## Abstract

Parvoviruses are a group of single-stranded DNA viruses that lack an envelope and are widely distributed in both vertebrates and invertebrates. When they infect a host cell, parvoviruses take over the cell’s translational machinery to support the viral genome replication and proteins synthesis, following which viral proteins undergo various post-translational modifications (PTMs). Parvovirus non-structural (NS) and capsid proteins are modified by PTMs, including phosphorylation, ubiquitination, SUMOylation, and glycosylation. Phosphorylation of parvovirus mainly occurs on NS and capsid proteins, modulating the functions and activities of the NS protein and the assembly of the capsid protein. Ubiquitination and SUMOylation of parvoviral capsid proteins mainly affect intracellular trafficking during viral infection. Glycosylation of parvoviral capsid proteins is involved in the regulation of virion stability and infectivity. In this review, we summarize the PTMs of parvovirus proteins and discuss their impact on the viral life cycle, which will help in understanding viral replication and pathogenesis.

## 1 Introduction

Parvoviruses are a group of non-enveloped, single-stranded DNA viruses characterized by an icosahedral capsid measuring 18–26 nm in diameter. They exhibit a broad host range, infecting both vertebrates and invertebrates (1). Taxonomically, the family *Parvoviridae* is divided into three subfamilies: *Parvovirinae, Densovirinae, and Hamaparvovirinae*, as well as the genus *Metalloincertoparvovirus* (2). The viral genome, which is approximately 5 kb in length, contains left and right open reading frames (ORFs) that encode viral nonstructural (NS) proteins [called Rep proteins in adeno-associated virus (AAV) and goose parvovirus (GPV)] and capsid proteins, respectively (3). The ORFs are flanked by 5′ and 3′ terminal hairpin structures, which are essential for the regulation of viral gene expression (4). However, owing to the restricted coding capacity of the parvovirus genome, parvoviruses heavily depend on host factors and cellular machinery to perform the viral replication process after invading host cells.

In parvoviruses, the capsid is responsible for the adsorption of receptors and enters cells via the clathrin-dependent endocytic pathway (5). Under the navigation of nuclear localization signals, the capsid is transported to the nucleus through the endosomal pathway to release the viral genome (6–8). Without a viral DNA polymerase, parvoviruses rely solely on DNA replication machinery within the nucleus (9). With the aid of host factors (10, 11), parvoviral precursor mRNA is transcribed to generate matured mRNA transcripts that encode NS and capsid proteins in the cytoplasm, where they undergo post-translational modifications (PTMs).

PTMs are crucial biochemical processes in eukaryotic cells, wherein specific enzymes catalyze the formation of covalent bonds on one or more amino acid residues of target proteins. Common PTMs, including acylation, glycosylation, methylation, phosphorylation, ubiquitination, and SUMOylation, play a pivotal role in regulating protein conformation, stability, activity, subcellular localization, and interactions with other proteins (12, 13). As intracellular parasites, viruses are modified by various PTMs during the viral infection process, which are involved in viral replication, assembly, release, and immune evasion. For instance, phosphorylation of VP8 in bovine herpesvirus 1 promotes viral DNA encapsidation in cells (14); glycosylation of the SARS-CoV-2 S protein shields its epitopes and enhances viral immune evasion (15). Similar to other viruses, PTMs also play an important role in the parvovirus life cycle. In this review, based on recent advances in PTM research on parvoviruses, we summarize the current knowledge of the PTMs of parvoviral proteins, including NS and capsid proteins. We also discuss the impact of PTMs on the parvoviral life cycle, which may help us better understand parvoviral replication and pathogenesis mechanisms.

## 2 Phosphorylation

### 2.1 NS protein

The large NS protein (NS1) of parvovirus is a multifunctional protein that possesses endonuclease, helicase, ATPase, and transcription-activating activities. These enzymatic activities are vital for initiating viral genome replication (16, 17) and regulating viral gene expression with the aid of host factors, which include the transcription factor Sp1/Sp3 and the TATA-binding protein (18, 19). However, its activities are heavily regulated by phosphorylation.

Phosphorylation of the parvoviral NS1 protein was first described in porcine parvovirus (PPV) in 1985 (20). Subsequent studies demonstrated that the Rep proteins of AAV, as well as the NS proteins of MVM and canine parvovirus (CPV), are also modified by phosphorylation (21–23). Biochemical activity analysis of MVM NS1 revealed that phosphorylation increased its viral helicase, ATPase, and nickase activities (24), while dephosphorylation led to a dramatic reduction in these enzymatic activities, suggesting that the replication functions of MVM NS1 are regulated by phosphorylation. Further analysis of phosphorylation site localization revealed that the helicase domain of MVM NS1 was phosphorylated, and the T363, T394, T403, T435, and S473 were identified as phosphorylation sites (25). The rat-origin protein kinase C *λ* was found to target T435 and S473 of MVM NS1 to phosphorylate it, resulting in the activation of its helicase function for initiating viral DNA unwinding and replication (26). In addition, PKC *η* could accelerate the phosphorylation of MVM NS1, which is necessary for the production of viral double-stranded concatemeric DNA intermediates during the early stage of virus replication (27).

The cytotoxicity of MVM NS1 is also regulated through phosphorylation. The parvovirus NS1 protein is the major effector that induces cytotoxicity in host cells (28–30). The C-terminus of MVM NS1 is identified as a major regulator of NS1 cytotoxicity (31). Further mutation analysis indicated that phosphorylation at the T585 site located at the C-terminus of MVM NS1 showed higher toxicity to A9 cells (28). In addition, the phosphorylation sites T598 and T601, located at the C-terminus of CPV NS1, were found to determine CPV pathogenicity (23). Phosphorylated MVM NS1 may exert its cytotoxic by controlling host gene expression and binding host proteins. Specifically, it can induce cytotoxicity by significantly activating thyroid hormone signaling pathways in FREJ4 cells (32), promoting oncogene expression in FR3T3 rat cells (33), and altering the synthesis of intracellular phosphorylated proteins (29). At the protein level, it also can interact with endogenous casein kinase II (CKII) to alter CKII’s activity, resulting in morphological and physiological alterations in host cells (34) and even cell lysis.

### 2.2 Capsid protein

Phosphorylation of viral structural proteins plays a crucial role at various stages of the viral life cycle, including recognition between intracellular host proteins and viruses, transport of viral proteins, capsid assembly, and genome packaging. For example, phosphorylation of the influenza virus nucleoprotein is vital for the assembly of ribonucleoprotein complexes (35). Phosphorylation of the core protein of hepatitis B virus is essential for the nuclear import of viral RNA and the stability of viral capsid proteins (36). Current research has found that the structural proteins of parvoviruses also undergo phosphorylation, which primarily regulates the assembly of capsid proteins.

In parvoviruses, the structural proteins include two or three capsid proteins (VP1-2 or VP1-3). Phosphorylation of parvoviral capsid proteins has been identified in MVM, PPV, and AAV (20, 37–39). MVM VP1 and VP2 are phosphorylated at serine and threonine residues located at the N-terminus in NB324K cells (37). The VP2 N-terminal sequence has been identified as the major phosphorylation domain harboring four phosphorylated residues including S2, S6, S10, and S16. Mutation analysis revealed that the major phosphorylation site S2 significantly affects the viral plaque-forming ability and plaque size, suggesting that it may primarily influence the viral budding or release process (37). Further research has indicated that the phosphorylated N-terminus of VP2 acts as a nuclear export signal, promoting the nuclear export of progeny viral particles during the replication process (40). In addition, phosphorylated residues on the viral particle surface are also required for this process (41). The progeny particles exported into the cytoplasm are further modulated through sequential processing in the endoplasmic reticulum and Golgi apparatus, where full infectivity of the viral particles is conferred (42).

Similar to nuclear export, the nuclear import of MVM capsid proteins also requires phosphorylation by the Raf-1 kinase in the cytoplasm (43, 44). The capsid proteins phosphorylated by the Raf-1 kinase enhance nuclear import and can be transported into the nucleus for completing progeny particle assembly (43). However, parental AAV capsids with tyrosine phosphorylation in the cytoplasm exhibit inefficient nuclear import ability (39), suggesting that this modification may trigger host defense mechanisms.

**Figure 1:**
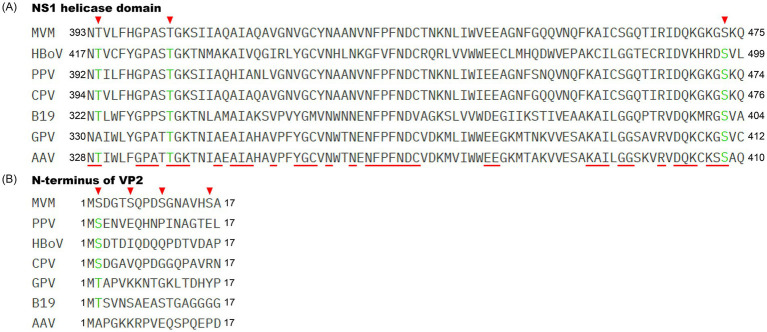
Schematic diagram showing the localization of potential phosphorylation sites in parvoviral NS1 helicase domain and the N-terminus of VP2. Alignment of the parvoviral helicase domain of the NS1 sequence **(A)** and the N-terminus of the VP2 sequence **(B)** The numbers on the left and right represent amino acid positions. The conserved residues in the helicase domain of parvovirus NS1 are highlighted with red lines. The identified phosphorylation sites in the MVM NS1 helicase domain and N-terminus of VP2 are indicated by the inverted red triangle. The potential phosphorylation sites are indicated in green font. The GenBank accession numbers for the NS1 or Rep and VP2 proteins of parvoviruses are as follows: the GenBank accession number for AAV2 Rep78 and VP2, CPV NS1 and VP2, HBoV NS1 and VP2, GPV Rep1 and VP2, PPV NS1 and VP2, and B19 NS1 and VP2 are DQ180605, DQ180605, KP710213, KC996729, AY684872 and AB030673, respectively; the GenBank accession number for MVM NS1 and VP2 are NP_041242 and J02275, respectively.

The NS1 proteins of parvoviruses share relatively high sequence homology across different genera, especially within the helicase domain, and perform similar functions in viral genome replication. Protein sequence alignment based on the helicase domain of MVM, CPV, PPV, parvovirus B19, AAV, human bocavirus (HBoV), and GPV (Figure 1A) showed that the phosphorylation sites of T394, T403, and S473 on MVM NS1 are conserved among these parvoviruses, suggesting that phosphorylation plays a critical role in the helicase activity of parvovirus NS1 and viral genome replication. Notably, the ser in the second site of VP2 is conserved among MVM, CPV, PPV, and HBoV (Figure 1B), meanwhile the tyr in the second site of GPV and B19 VP2 may serve as a potential phosphorylation site, indicating that phosphorylation also governs the nuclear import and export dynamics of viral progeny proteins, facilitating viral maturation and release.

## 3 Ubiquitination and SUMOylation

Ubiquitination of parvoviral capsid proteins was first identified in AAV type 2 (AAV2) in 2000 (45). Using liquid chromatography and mass spectrometry analysis, some conserved ubiquitination sites on the AAV capsid surface were found in AAV4, AAV5, AAV9, and AAVrh10 (38, 46). These findings suggest that ubiquitination of viral capsid proteins may affect the parvoviral life cycle. In AAV2-infected cells, the viral intracellular trafficking is enhanced by treating cells with proteasome inhibitor MG132 (46, 47). Furthermore, mutation of ubiquitination sites in the AAV2 capsid protein, such as K490, K544, K549, and K55, significantly promoted the transduction efficiency of AAV2 both *in vitro* and *in vivo* (48–50). In addition, inhibition of the EGFR-PTK signaling pathway mediating ubiquitination of the AAV2 capsid protein also increased the trafficking efficiency of AAV2 (51). In contrast, the ubiquitin–proteasome pathway is required for the replication of MVM and CPV. Inhibiting the ubiquitin–proteasome pathway using MG132 in MVM- and CPV-infected cell abolished MVM and CPV replication (52), which is similar to what has been observed with human immunodeficiency virus and Rous sarcoma virus (53, 54). In addition to parvoviral capsid proteins, MVM NS2 and AAV Rep proteins also undergo ubiquitination modification and subsequent proteasomal degradation (55–57). However, the specific mechanism between the degradation of NS2 or Rep proteins via the proteasomal degradation pathway and viral replication remains to be elucidated.

In addition to ubiquitination, both the Rep and capsid proteins of AAV2 undergo SUMOylation (38, 58). SUMOylation of AAV2 Rep78 has been demonstrated to regulate its stability and extend its half-life (58). Similar to ubiquitination, SUMOylation of AAV capsid proteins has been shown to influence the efficiency of intracellular transduction (38). Inhibition of the SUMOylation pathway in AAV2-infected cells resulted in increased transduction efficiency within the cells (59). Furthermore, a systematic screening method using an siRNA library was employed to identify host factors regulating viral replication. This screening revealed that multiple host factors involved in the SUMOylation signaling pathway regulate AAV infection (60). Further knockdown of the SUMOylation components Ubc9 and Sae2 significantly enhanced AAV2 transduction. Mechanistic studies have revealed that AAV infection activates the intracellular SUMOylation machinery, which subsequently restricts AAV transduction efficiency (61).

As a delivery vehicle for gene therapy, the AAV vector cannot encode viral proteins to antagonize host restriction factors inherent to cellular defense mechanisms, since the AAV vector maintains a simplified structure composed solely of an exogenous therapeutic genome packaged within capsid proteins. Therefore, further optimization of the vector’s ubiquitination and SUMOylation target sites could improve delivery efficiency.

## 4 Glycosylation

Glycosylation is one of the most crucial post-translational modifications and regulates enveloped virus infection by mediating receptor binding, facilitating protein folding and trafficking, and participating in immune evasion (62, 63). However, there is very little information on parvoviral protein glycosylation. To date, glycosylation has been detected only in the capsid proteins of AAV2, AAV3, AAV5, AAV7, AAV8, AAV9, and AAVrh10using liquid chromatography and mass spectrometry analysis (38, 64, 65). Specifically, residue N499 of the AAV8 capsid protein and residue N253, N518, S537, and N551 of the AAV2 capsid protein have been identified as N-linked glycosylation sites (38, 64). In addition, both N- and O-linked glycosylation sites have been detected in the AAV9 capsid protein (65). Mutation analysis revealed that the AAV2 capsid protein with the N253Q mutation resulted in a considerable reduction in vector yield compared to AAV2 wild-type vectors, suggesting that glycosylation of the capsid is critical for virion assembly and stability (64). On the other hand, a number of potential glycosylation sites in the capsid protein of GPV, Muscovy duck parvovirus, and Aleutian mink disease virus have been predicted based on bioinformatic analyses of the capsid protein sequences (66, 67). These studies suggest that capsid protein glycosylation may be a conserved feature among all parvoviruses. However, the molecular mechanism by which parvoviral capsid glycosylation modulates viral tissue tropism, intracellular trafficking, infectivity, and immunogenicity remains to be further studied.

To date, glycosylation of NS proteins in parvovirus has not been reported. Whether the NS proteins of parvovirus possess glycosylation sites and whether these modifications affect NS proteins’ function and activity are yet to be uncovered.

## 5 Discussion

As an intracellular parasite, parvovirus relies heavily on a variety of cellular mechanisms to accomplish its life cycle. During parvoviral infection, parvovirus proteins undergo phosphorylation, ubiquitination, SUMOylation, and glycosylation (Table 1). Glycosylation has been detected in parvovirus capsid proteins and may regulate virion stability, receptor binding, immunogenicity, and other functions. Meanwhile, phosphorylation not only regulates the NS1 protein’s function activity and cytotoxicity but also promotes the assembly and maturation of progeny virions. Ubiquitination and SUMOylation restrict parvovirus infection, resulting in inefficient intracellular trafficking of viral capsids within cells. Nevertheless, how other PTMs—including, but not limited to, lipidation and methylation—modulate parvoviral replication dynamics remains to be fully characterized. Parvoviruses are promising vectors for gene therapy and oncolytic virotherapy. Further studies on parvoviral PTMs will contribute to the development of novel bioengineered gene therapy vectors and novel inhibitors.

**Table 1:** Effects of PTMs of parvoviral proteins on the virus.

Modification	Related protein	Impacts on the virus	Reference
Phosphorylation	PPV NS1	Unknown	20
	MVM NS1	Regulates the NS protein’s enzymatic activity, cytotoxicity, and attachment to viral DNA	22, 24, 25, 31
	AAV Rep	Promotes viral DNA synthesis	23
	MVM VP1 and VP2	Facilitates viral maturation and release	37, 40, 41, 43, 44
	AAV capsid proteins	Inhibits the intracellular trafficking process	38, 39
Ubiquitination	AAV capsid proteins	Inhibits the intracellular trafficking process	38, 45–47
	MVM NS2	Degrades viral NS2	55
	AAV Reps	Degrades viral Rep proteins	56, 57
SUMOylation	AAV2 Rep78	Regulates the Rep78 protein’s stability and half-life	58
	AAV2 capsid proteins	Inhibits the intracellular trafficking process	38, 59
Glycosylation	AAV capsid proteins	May regulate virion stability and infectivity.	38, 64, 65

AAV Reps represent the viral proteins Rep78, Rep52, and Rep40.

To counteract viral infection, host cells deploy a broad spectrum of antiviral defenses, including interferon-induced antiviral factors and cell-intrinsic restriction factors, which directly target viral proteins or genomes. However, type I interferon signaling fails to effectively suppress infections by autonomous parvoviruses and AAV2 in both normal human and cancer cells (68). Cell-intrinsic restriction factors likely play an important role in the host’s defense against parvoviruses. Systematic screening of these antiviral factors, coupled with mechanistic studies of their viral targets, will provide a solid foundation for the development of gene therapy vectors with improved safety and efficacy.

## References

[1] Cotmore SF Tattersall P. Parvoviral host range and cell entry mechanisms. Adv Virus Res. (2007) 70:183–232. doi: 10.1016/S0065-3527(07)70005-2, PMID: 17765706

[2] Kibenge F Kibenge M Montes De Oca M Godoy M. Parvoviruses of aquatic animals. Pathogens. (2024) 13:625. doi: 10.3390/pathogens13080625, PMID: 39204226 PMC11357303

[3] Cotmore SF Tattersall P. The autonomously replicating parvoviruses of vertebrates. Adv Virus Res. (1987) 33:91–174. doi: 10.1016/s0065-3527(08)60317-6 3296697

[4] Wang XS Ponnazhagan S Srivastava A. Rescue and replication of adeno-associated virus type 2 as well as vector DNA sequences from recombinant plasmids containing deletions in the viral inverted terminal repeats: selective encapsidation of viral genomes in progeny virions. J Virol. (1996) 70:1668–77. doi: 10.1128/JVI.70.3.1668-1677.1996, PMID: 8627687 PMC189990

[5] Parker JSL Parrish CR. Cellular uptake and infection by canine parvovirus involves rapid dynamin-regulated clathrin-mediated endocytosis, followed by slower intracellular trafficking. J Virol. (2000) 74:1919–30. doi: 10.1128/jvi.74.4.1919-1930.2000, PMID: 10644365 PMC111670

[6] Harbison CE Chiorini JA Parrish CR. The parvovirus capsid odyssey: from the cell surface to the nucleus. Trends Microbiol. (2008) 16:208–14. doi: 10.1016/j.tim.2008.01.012, PMID: 18406140

[7] Sonntag F Bleker S Leuchs B Fischer R Kleinschmidt JA. Adeno-associated virus type 2 capsids with externalized VP1/VP2 trafficking domains are generated prior to passage through the cytoplasm and are maintained until uncoating occurs in the nucleus. J Virol. (2006) 80:11040–54. doi: 10.1128/JVI.01056-06, PMID: 16956943 PMC1642181

[8] Liu P Chen S Wang M Cheng A. The role of nuclear localization signal in parvovirus life cycle. Virol J. (2017) 14:80. doi: 10.1186/s12985-017-0745-1, PMID: 28410597 PMC5391597

[9] Qiu J Söderlund-Venermo M Young NS. Human Parvoviruses. Clin Microbiol Rev. (2017) 30:43–113. doi: 10.1128/CMR.00040-16, PMID: 27806994 PMC5217800

[10] Raab U Bauer B Gigler A Beckenlehner K Wolf H Modrow S. Cellular transcription factors that interact with p6 promoter elements of parvovirus B19. J Gen Virol. (2001) 82:1473–80. doi: 10.1099/0022-1317-82-6-1473, PMID: 11369893

[11] Momoeda M Kawase M Jane SM Miyamura K Young NS Kajigaya S. The transcriptional regulator YY1 binds to the 5′-terminal region of B19 parvovirus and regulates P6 promoter activity. J Virol. (1994) 68:7159–68. doi: 10.1128/JVI.68.11.7159-7168.1994, PMID: 7933098 PMC237155

[12] Lee JM Hammarén HM Savitski MM Baek SH. Control of protein stability by post-translational modifications. Nat Commun. (2023) 14:201. doi: 10.1038/s41467-023-35795-8, PMID: 36639369 PMC9839724

[13] Tay AP Liang A Wilkins MR Pang CNI. Visualizing post-translational modifications in protein interaction networks using PTMOracle. Curr Protoc Bioinformatics. (2019) 66:e71. doi: 10.1002/cpbi.71, PMID: 30653846

[14] Kuan Z Robert B Marlene S Sylvia LVDH. Phosphorylation of bovine herpesvirus 1 VP8 plays a role in viral DNA encapsidation and is essential for its cytoplasmic localization and optimal virion incorporation. J Virol. (2016) 90:4427–40. doi: 10.1128/JVI.00219-16 26889039 PMC4836321

[15] Zhao P Praissman JL Grant OC Cai Y Xiao T Rosenbalm KE. Virus-receptor interactions of glycosylated sars-Cov-2 spike and human Ace2 receptor. Cell Host Microbe. (2020) 28:586–601.e6. doi: 10.1016/j.chom.2020.08.004, PMID: 32841605 PMC7443692

[16] Jindal HK Yong CB Wilson GM Tam P Astell CR. Mutations in the NTP-binding motif of minute virus of mice (MVM) NS-1 protein uncouple ATPase and DNA helicase functions. J Biol Chem. (1994) 269:3283–9. doi: 10.1016/S0021-9258(17)41860-6, PMID: 8106366

[17] Wilson GM Jindal HK Yeung DE Chen W Astell CR. Expression of minute virus of mice major nonstructural protein in insect cells: purification and identification of ATPase and helicase activities. Virology. (1991) 185:90–8. doi: 10.1016/0042-6822(91)90757-3, PMID: 1833878

[18] Raab U Beckenlehner K Lowin T Niller HH Doyle S Modrow S. NS1 protein of parvovirus b19 interacts directly with DNA sequences of the p6 promoter and with the cellular transcription factors sp1/sp3. Virology. (2002) 293:86–93. doi: 10.1006/viro.2001.1285, PMID: 11853402

[19] Francois A Guilbaud M Awedikian R Chadeuf G Moullier P Salvetti A. The cellular TATA binding protein is required for rep-dependent replication of a minimal adeno-associated virus type 2 p5 element. J Virol. (2005) 79:11082–94. doi: 10.1128/JVI.79.17.11082-11094.2005, PMID: 16103159 PMC1193596

[20] Molitor TW Joo HS Collett MS. Identification and characterization of a porcine parvovirus nonstructural polypeptide. J Virol. (1985) 55:554–9. doi: 10.1128/JVI.55.3.554-559.1985, PMID: 4020958 PMC255006

[21] Cotmore SF Tattersall P. The NS-1 polypeptide of the autonomous parvovirus MVM is a nuclear phosphoprotein. Virus Res. (1986) 4:243–50. doi: 10.1016/0168-1702(86)90003-1, PMID: 3739422

[22] Collaco R Prasad KMR Trempe JP. Phosphorylation of the adeno-associated virus replication proteins. Virology. (1997) 232:332–6. doi: 10.1006/viro.1997.8563, PMID: 9191846

[23] Miao B Chen S Zhang X Ma P Ma M Chen C. T598 and T601 phosphorylation sites of canine parvovirus NS1 are crucial for viral replication and pathogenicity. Vet Microbiol. (2022) 264:109301. doi: 10.1016/j.vetmic.2021.109301, PMID: 34915313

[24] Nüesch JPF Corbau R Tattersall P Rommelaere J. Biochemical activities of minute virus of mice nonstructural protein NS1 are modulated in vitro by the phosphorylation state of the polypeptide. J Virol. (1998) 72:8002–12. doi: 10.1128/JVI.72.10.8002-8012.1998, PMID: 9733839 PMC110136

[25] Corbau R Salomé N Rommelaere J Nüesch JPF. Phosphorylation of the viral nonstructural protein NS1 during MVMp infection of a9 cells. Virology. (1999) 259:402–15. doi: 10.1006/viro.1999.9786, PMID: 10388664

[26] Nuesch JPF Christensen J Rommelaere J. Initiation of minute virus of mice DNA replication is regulated at the level of origin unwinding by atypical protein kinase c phosphorylation of NS1. J Virol. (2001) 75:5730–9. doi: 10.1128/JVI.75.13.5730-5739.2001, PMID: 11390575 PMC114289

[27] Lachmann S Rommeleare J Nuesch JPF. Novel PKCη is required to activate replicative functions of the major nonstructural protein NS1 of minute virus of mice. J Virol. (2003) 77:8048–60. doi: 10.1128/jvi.77.14.8048-8060.2003 12829844 PMC161934

[28] Daeffler L Hörlein R Rommelaere J Nüesch JPF. Modulation of minute virus of mice cytotoxic activities through site-directed mutagenesis within the NS coding region. J Virol. (2003) 77:12466–78. doi: 10.1128/jvi.77.23.12466-12478.2003, PMID: 14610171 PMC262581

[29] Anouja F Wattiez R Mousset S Caillet-Fauquet P. The cytotoxicity of the parvovirus minute virus of mice nonstructural protein NS1 is related to changes in the synthesis and phosphorylation of cell proteins. J Virol. (1997) 71:4671–8. doi: 10.1128/JVI.71.6.4671-4678.1997, PMID: 9151861 PMC191689

[30] Corbau R Duverger V Rommelaere J Nüesch JP. Regulation of MVM NS1 by protein kinase c: impact of mutagenesis at consensus phosphorylation sites on replicative functions and cytopathic effects. Virology. (2000) 278:151–67. doi: 10.1006/viro.2000.0600, PMID: 11112491

[31] Legendre D Rommelaere J. Terminal regions of the NS-1 protein of the parvovirus minute virus of mice are involved in cytotoxicity and promoter trans inhibition. J Virol. (1992) 66:5705–13. doi: 10.1128/JVI.66.10.5705-5713.1992, PMID: 1388209 PMC241445

[32] Vanacker JM Laudet V Adelmant G Stéhelin D Rommelaere J. Interconnection between thyroid hormone signalling pathways and parvovirus cytotoxic functions. J Virol. (1993) 67:7668–72. doi: 10.1128/JVI.67.12.7668-7672.1993, PMID: 8230488 PMC238238

[33] Mousset S Rommelaere J. The cytotoxicity of the autonomous parvovirus minute virus of mice nonstructural proteins in FR3t3 rat cells depends on oncogene expression. J Virol. (1994) 68:6446–53. doi: 10.1128/JVI.68.10.6446-6453.1994, PMID: 8083981 PMC237064

[34] Nüesch JP Rommelaere J. A viral adaptor protein modulating casein kinase II activity induces cytopathic effects in permissive cells. Proc Natl Acad Sci USA. (2007) 104:12482–7. doi: 10.1073/pnas.0705533104, PMID: 17636126 PMC1920537

[35] Mondal A Potts GK Dawson AR Coon JJ Mehle A. Phosphorylation at the homotypic interface regulates nucleoprotein oligomerization and assembly of the influenza virus replication machinery. PLoS Pathog. (2015) 11:e1004826. doi: 10.1371/journal.ppat.1004826, PMID: 25867750 PMC4395114

[36] Lubyov B Weber J. Posttranslational modifications of HBV core protein. Acta Virol. (2020) 64:177–86. doi: 10.4149/av_2020_207 32551786

[37] Maroto B Jc R Almendral JM. Phosphorylation status of the parvovirus minute virus of mice particle: mapping and biological relevance of the major phosphorylation sites. J Virol. (2000) 74:10892–902. doi: 10.1128/jvi.74.23.10892-10902.2000 11069983 PMC113168

[38] Mary B Maurya S Arumugam S Kumar V Jayandharan GR. Post-translational modifications in capsid proteins of recombinant adeno-associated virus (AAV) 1-rh10 serotypes. FEBS J. (2019) 286:4964–81. doi: 10.1111/febs.15013, PMID: 31330090 PMC7496479

[39] Zhong L Li B Jayandharan G Mah CS Govindasamy L Agbandje-Mckenna M. Tyrosine-phosphorylation of AAV2 vectors and its consequences on viral intracellular trafficking and transgene expression. Virology. (2008) 381:194–202. doi: 10.1016/j.virol.2008.08.027, PMID: 18834608 PMC2643069

[40] Maroto B Valle N Saffrich R Almendral JM. Nuclear export of the nonenveloped parvovirus virion is directed by an unordered protein signal exposed on the capsid surface. J Virol. (2004) 78:10685–94. doi: 10.1128/JVI.78.19.10685-10694.2004, PMID: 15367635 PMC516424

[41] Wolfisberg R Kempf C Ros C. Late maturation steps preceding selective nuclear export and egress of progeny parvovirus. J Virol. (2016) 90:5462–74. doi: 10.1128/JVI.02967-15, PMID: 27009963 PMC4934750

[42] Bär S Rommelaere J Nüesch JPF Weitzman MD. Vesicular transport of progeny parvovirus particles through ER and golgi regulates maturation and cytolysis. PLoS Pathog. (2013) 9:e1003605. doi: 10.1371/journal.ppat.1003605 24068925 PMC3777860

[43] Riolobos L Valle N Hernando E Maroto B Kann M Almendral JM. Viral oncolysis that targets raf-1 signaling control of nuclear transport. J Virol. (2010) 84:2090–9. doi: 10.1128/JVI.01550-09, PMID: 19939915 PMC2812376

[44] Gil-Ranedo J Hernando E Valle N Riolobos L Maroto B Almendral JM. Differential phosphorylation and N-terminal configuration of capsid subunits in parvovirus assembly and viral trafficking. Virology. (2018) 518:184–94. doi: 10.1016/j.virol.2018.02.018, PMID: 29524834

[45] Duan D Yue Y Yan Z Yang J Engelhardt JF. Endosomal processing limits gene transfer to polarized airway epithelia by adeno-associated virus. J Clin Invest. (2000) 105:1573–87. doi: 10.1172/JCI8317, PMID: 10841516 PMC300848

[46] Yan Z Zak R Luxton GWG Ritchie TC Bantel-Schaal U Engelhardt JF. Ubiquitination of both adeno-associated virus type 2 and 5 capsid proteins affects the transduction efficiency of recombinant vectors. J Virol. (2002) 76:2043–53. doi: 10.1128/jvi.76.5.2043-2053.2002, PMID: 11836382 PMC135943

[47] Douar AM Poulard K Stockholm D Danos O. Intracellular trafficking of adeno-associated virus vectors: routing to the late endosomal compartment and proteasome degradation. J Virol. (2001) 75:1824–33. doi: 10.1128/JVI.75.4.1824-1833.2001, PMID: 11160681 PMC114092

[48] Gabriel N Hareendran S Sen D Gadkari RA Sudha G Selot R. Bioengineering of AAV2 capsid at specific serine, threonine, or lysine residues improves its transduction efficiency in vitro and in vivo. Hum Gene Ther Clin Dev. (2013) 24:80–93. doi: 10.1089/hgtb.2012.194, PMID: 23379478 PMC3732126

[49] Mao Y Wang X Yan R Hu W Li A Wang S. Single point mutation in adeno-associated viral vectors -DJ capsid leads to improvement for gene delivery in vivo. BMC Biotechnol. (2016) 16:1–8. doi: 10.1186/s12896-015-0230-0, PMID: 26729248 PMC4700607

[50] Srivastava A Li B Ma W Ling C Vliet KV Huang L. Site-directed mutagenesis of surface-exposed lysine residues leads to improved transduction by AAV2, but not AAV8 vectors in murine hepatocytes in vivo. Hum Gene Ther Methods. (2015) 26:211–20. doi: 10.1089/hgtb.2015.115 26421998 PMC4677520

[51] Zhong L Zhao W Wu J Li B Srivastava A. A dual role of EGFR protein tyrosine kinase signaling in ubiquitination of AAV2 capsids and viral second-strand DNA synthesis. Mol Ther. (2007) 15:1323. doi: 10.1038/sj.mt.6300170, PMID: 17440440

[52] Ros C Burckhardt CJ Kempf C. Cytoplasmic trafficking of minute virus of mice: low-ph requirement, routing to late endosomes, and proteasome interaction. J Virol. (2002) 76:12634–45. doi: 10.1128/jvi.76.24.12634-12645.2002, PMID: 12438589 PMC136711

[53] Schubert U Ott DE. Proteasome inhibition interferes with gag polyprotein processing, release, and maturation of HIV-1 and HIV-2. Proc Natl Acad Sci USA. (2000) 97:13057. doi: 10.1073/pnas.97.24.13057, PMID: 11087859 PMC27177

[54] Vana ML Tang Y Chen A Medina G Carter C Leis J. Role of nedd4 and ubiquitination of rous sarcoma virus gag in budding of virus-like particles from cells. J Virol. (2004) 78:13943–53. doi: 10.1128/JVI.78.24.13943-13953.2004, PMID: 15564502 PMC533940

[55] Miller CL Pintel DJ. The NS2 protein generated by the parvovirus minute virus of mice is degraded by the proteasome in a manner independent of ubiquitin chain elongation or activation. Virology. (2001) 285:346–55. doi: 10.1006/viro.2001.0966, PMID: 11437668

[56] Sukhu L Pintel D. The large rep protein of adeno-associated virus type 2 is polyubiquitinated. J Gen Virol. (2011) 92:2792–6. doi: 10.1099/vir.0.034975-0, PMID: 21865444 PMC3352567

[57] Farris KD Fasina O Sukhu L Li L Pintel DJ. Adeno-associated virus small rep proteins are modified with at least two types of polyubiquitination. J Virol. (2010) 84:1206–11. doi: 10.1128/JVI.01660-09, PMID: 19889761 PMC2798352

[58] Weger S Hammer E Heilbronn R. SUMO-1 modification regulates the protein stability of the large regulatory protein rep78 of adeno associated virus type 2 (AAV-2). Virology. (2004) 330:284–94. doi: 10.1016/j.virol.2004.09.028, PMID: 15527853

[59] Maurya S Jayandharan GR. Exosome-associated SUMOylation mutant AAV demonstrates improved ocular gene transfer efficiency in vivo. Virus Res. (2020) 283:197966. doi: 10.1016/j.virusres.2020.197966, PMID: 32302639 PMC7212041

[60] Hlscher C Sonntag F Henrich K Chen Q Müller M. The SUMOylation pathway restricts gene transduction by adeno-associated viruses. PLoS Pathog. (2015) 11:e1005281. doi: 10.1371/journal.ppat.1005281 26625259 PMC4666624

[61] Chen Q Njenga R Leuchs B Chiocca S Müller M. Sumoylation targets AAV capsids but mainly restricts transduction by cellular mechanisms. J Virol. (2020) 94:e00871-20. doi: 10.1128/JVI.00871-20 32669341 PMC7495375

[62] Goffard A Dubuisson J. Glycosylation of hepatitis c virus envelope proteins. Biochimie. (2003) 85:295–301. doi: 10.1016/s0300-9084(03)00004-x, PMID: 12770768

[63] Feng T Zhang J Chen Z Pan W Chen Z Yan Y. Glycosylation of viral proteins: implication in virus-host interaction and virulence. Virulence. (2022) 13:670–83. doi: 10.1080/21505594.2022.2060464, PMID: 35436420 PMC9037552

[64] Aloor A Zhang J Gashash E Parameswaran A Xiao W. Site-specific n-glycosylation on the AAV8 capsid protein. Viruses. (2018) 10:644. doi: 10.3390/v10110644, PMID: 30453606 PMC6266768

[65] Zhou Y Priya S Ong JY. Characterizing glycosylation of adeno-associated virus serotype 9 capsid proteins generated from HEK293 cells through Glycopeptide mapping and released glycan analysis. Microorganisms. (2024) 12:946. doi: 10.3390/microorganisms12050946, PMID: 38792776 PMC11123743

[66] Liu HM Wang H Tian XJ Zhang S Zhou XH Qi KZ. Complete genome sequence of goose parvovirus Y strain isolated from Muscovy ducks in China. Virus Genes. (2014) 48:199–202. doi: 10.1007/s11262-013-1001-4, PMID: 24194370

[67] Li Y Huang J Jia Y Du Y Jiang P Zhang R. Genetic characterization of Aleutian mink disease viruses isolated in China. Virus Genes. (2012) 45:24–30. doi: 10.1007/s11262-012-0733-x, PMID: 22415541

[68] Paglino JC Andres W van den Pol AN. Autonomous parvoviruses neither stimulate nor are inhibited by the type I interferon response in human normal or cancer cells. J Virol. (2014) 88:4932–42. doi: 10.1128/JVI.03508-13, PMID: 24554651 PMC3993814

